# Effects of break crops, and of wheat volunteers growing in break crops or in set-aside or conservation covers, all following crops of winter wheat, on the development of take-all (*Gaeumannomyces graminis* var. *tritici*) in succeeding crops of winter wheat

**DOI:** 10.1111/aab.12139

**Published:** 2014-06-11

**Authors:** JF Jenkyn, RJ Gutteridge, RP White

**Affiliations:** Rothamsted ResearchHarpenden, UK

**Keywords:** Break crops, set-aside, take-all, volunteers, wheat

## Abstract

Experiments on the Rothamsted and Woburn Experimental Farms studied the effects on take-all of different break crops and of set-aside/conservation covers that interrupted sequences of winter wheat. There was no evidence for different effects on take-all of the break crops *per se* but the presence of volunteers, in crops of oilseed rape, increased the amounts of take-all in the following wheat. Severity of take-all was closely related to the numbers of volunteers in the preceding break crops and covers, and was affected by the date of their destruction. Early destruction of set-aside/conservation covers was usually effective in preventing damaging take-all in the following wheat except, sometimes, when populations of volunteers were very large. The experiments were not designed to test the effects of sowing dates but different amounts of take-all in the first wheats after breaks or covers apparently affected the severity of take-all in the following (second) wheats only where the latter were relatively late sown. In earlier-sown second wheats, take-all was consistently severe and unrelated to the severity of the disease in the preceding (first) wheats. Results from two very simple experiments suggested that substituting set-aside/conservation covers for winter wheat, for 1 year only, did not seriously interfere with the development of take-all disease or with the development or maintenance of take-all decline (TAD). With further research, it might be possible for growers wishing to exploit TAD to incorporate set-aside/conservation covers into their cropping strategies, and especially to avoid the worst effects of the disease on grain yield during the early stages of epidemics.

## Introduction

Take-all (caused by *Gaeumannomyces graminis* var. *tritici*; Ggt*)* is a potentially damaging disease of wheat roots in most parts of the world where the crop is grown. The fungus does not, however, survive well in the soil in the absence of its hosts, which are almost exclusively graminaceous species, including barley and other temperate cereals, except oats, and some grasses. Consequently, take-all is seldom damaging in wheat crops that follow non-graminaceous break crops. If, however, susceptible cereals are grown consecutively, take-all typically increases to damaging severities in the second to fourth crops. It follows that losses due to take-all can be almost completely avoided by adopting ‘good’ rotations. Many farmers, however, can grow only a limited range of break crops profitably because of unsuitable soils, distance from processing facilities or other factors, and, therefore, have to grow at least some cereal crops consecutively.

Generally, a 1-year, non-cereal break crop is sufficient to prevent significant yield losses due to take-all in the following first wheats. Different break crops may, however, differ in their capacity to maintain the take-all fungus through the break year, and hence in the potential they create for the disease to increase to damaging proportions in the following (i.e. second or subsequent) wheat crops. There is, for example, a perception that second wheats after oilseed rape (OSR) are often at greater risk from damaging take-all than second wheats after other breaks. This is, perhaps, further suggested by the increasing frequencies of reports of severe take-all in second wheats in the UK in the 1980s that coincided with dramatic increases in the areas of OSR (Hornby *et al*., 1998).

Despite this, there are, as far as we are aware, no published empirical data to suggest that there is a real and consistent difference between OSR and other break crops in their effects on take-all. Hornby *et al*. (1998) did, however, list a number of factors, acting alone or in combination, and operating principally through effects on the build-up of take-all inoculum in first wheats, that could cause such a difference. First, wheat after OSR, and especially after winter-sown OSR, is usually sown much earlier than wheat after other breaks, which means that the take-all fungus has longer to multiply in the first wheat, thus posing a potentially bigger threat to the second wheat. Second, there is often more residual nitrogen in the soil after OSR than after many other break crops, sometimes including legumes (McEwen *et al*., 1990), and this can be expected to favour the saprophytic survival of the fungus in the soil (Garrett, 1938). Third, crops of OSR that interrupt a sequence of cereals (i.e. that follow a cereal) typically contain large populations of cereal volunteers on which Ggt can survive during the break year.

All three of the factors listed in the preceding paragraph could also, in theory, influence the relative extent to which Ggt can survive and multiply during and after non-graminaceous break crops apart from OSR. Another possibility, perhaps more specific to OSR among non-graminaceous species, is that Ggt might, to a limited extent, be able to infect its roots. Kollmorgen *et al*. (1983) were able to isolate the pathogen from lesions on the roots of OSR grown in pot experiments, a result that was confirmed by Hornby & Davis (1985) working in the UK. The latter authors found, however, that the number of roots infected and the proportion from which the fungus could be isolated were very small, leading them to conclude that ‘the take-all fungus does not appear to colonise rape roots readily’.

The threat posed by cereal volunteers is illustrated by the results of an experiment reported by Gutteridge & Hornby (2003), which showed that take-all in a late-sown crop of winter wheat that followed winter wheat was more severe where wheat volunteers were present in the inter-crop period than where they were controlled. Similar results, but from a glasshouse experiment, were previously reported by Brassett & Gilligan (1990). It can, therefore, be assumed that cereal volunteers growing in break crops have the potential to aid the survival and multiplication of Ggt, and thus increase the risk of damaging take-all in following susceptible crops. This may be especially so for OSR because where it follows a cereal it is often sown after non-inversion tillage, which can result in very large populations of volunteers. Controlling them is often recommended (e.g. Mielke, 1988) but, because OSR is such a vigorous crop, there may be a temptation to assume that competition from the OSR is likely to limit the growth of the volunteers and the risk that they pose. Controlling volunteers in OSR may not, therefore, be considered by some growers to be as important as in other breaks.

The risk posed by volunteers is likely to depend on how numerous they are, and whether or not they are controlled; probably also on how long they are allowed to grow before they are controlled and the interval between doing that and sowing the next susceptible crop. If so, they should pose a particular threat where a sequence of take-all susceptible cereals is interrupted by set-aside or conservation covers, where populations of volunteers can be very large, especially if they are established by natural regeneration, and where there are usually restrictions on how, and how early, they can be controlled. Results from previously reported experiments confirmed that take-all in winter wheat is typically more severe where volunteers in a preceding set-aside cover are allowed to establish and persist through the winter and spring than where they are prevented from establishing (Jenkyn *et al*., 1998) but provided no information on the relationship between numbers of volunteers (i.e. population density) and the risk that they pose nor on the effects of destroying volunteers at different times.

Although the severity of take-all typically increases in successive susceptible cereal crops after a break, a characteristic feature of the disease is the phenomenon known as take-all decline (TAD). This develops after a severe outbreak of disease in consecutive susceptible cereals, and is a consequence of natural biological control (Hornby *et al*., 1998). Take-all decline evidently occurs very widely, and crops grown in fields with well-established TAD typically have less severe take-all and, more importantly, larger yields than crops grown in the same fields when the disease was at its peak. Although yields are unlikely to match those obtained from first wheats after breaks, there are some growers who find that, in certain situations, it can make good commercial sense to exploit TAD by growing continuous cereals (e.g. wheat). Typically, such cropping is adopted in outlying fields or in fields with difficult access or with intractable soils that limit alternative cropping options. For such growers, it is important to know if effective and reliable TAD, possibly generated by many years of continuous cropping, is put at risk by interrupting a long run of cereals with 1-year set-aside. Equally, it would be useful to know if it is possible to generate effective TAD where set-aside or a conservation cover is substituted for a cereal crop in the year(s) when take-all is expected to be at its most severe thus avoiding the worst effects of the disease on yield, which are, otherwise, unavoidable.

In this article we describe field experiments designed to improve understanding of the effects of non-cereal break crops, and, especially, of wheat volunteers growing in break crops or in set-aside/conservation covers, on the development of take-all in subsequent crops of winter wheat. The first two experiments compared the effects on take-all of interrupting sequences of winter wheat with OSR or other break crops. Cereal volunteers were, as far as possible, controlled, and the following (first) wheats were all sown at the same time so that direct effects of the break crops would be the most likely explanation for any differences that might be detected. All of the other experiments that we describe were designed to study the effects of cereal volunteers either in an OSR break crop (two experiments) or in set-aside/conservation covers, all of which also interrupted sequences of winter wheat. In three of the latter, covers were established by natural regeneration but numbers of volunteers were very variable. In the last four experiments, wheat was, therefore, sown (in combination with mustard) to simulate volunteers as it also was in the OSR break crops. The experiments also tested the effects of controlling the volunteers in the OSR, and of destroying set-aside/conservation covers, at different times. Effects were measured in two or three consecutive crops of winter wheat that followed these treatments. Some of the results from the experiments in which covers were established by natural regeneration have been reported previously in conference proceedings (Jenkyn *et al*., 1996) but not in a peer-reviewed journal or in as much detail.

## Materials and methods

### Experiments 1 and 2

Each of these experiments followed two crops of winter wheat, and compared five different non-graminaceous break crops plus oats, which are not a host of Ggt, and wheat, all followed by another two crops of winter wheat that were treated uniformly. They were tested in four complete randomised blocks, giving a total of 28 plots in each experiment. The crops grown in the treatment years (1999 and 2000, respectively; years refer to year of harvest unless otherwise indicated) were all winter sown. They were: OSR (cv. Apex, sown 27 August 1998 and 1 September 1999); linseed (cv. Oliver, 12 and 6 October); lupins (cv. DTN 20, 7 September and DTN 12, 13 September); beans (cv. Clipper, 15 and 28 October); peas (cv. Victor, 15 and 29 October); oats (cv. Gerald, 16 and 6 October); and wheat (cv. Hereward, 21 and 17 September). Establishment in one of the wheat plots in Experiment 1 was, however, poor, and it had to be re-drilled on 13 October. The straw of the preceding wheat crops was baled and removed before the sites of the experiments were ploughed, to minimise numbers of wheat volunteers, and the treatment crops sown in plots that were 10 m long and 6 m wide. Applications of fertilisers and crop protection chemicals were appropriate for the different crops, and included, where necessary and where possible, herbicides to control wheat volunteers (except in plots of winter wheat).

After the treatment crops had been harvested, the straw was baled and removed, and the sites ploughed before sowing the first (i.e. 2000 and 2001) test crops of winter wheat (cv. Hereward) at 380 seeds m^−2^ on 17 September and at 250 seeds m^−2^ on 21 September in Experiments 1 and 2, respectively. Second test crops of winter wheat were, similarly, sown after the straw of the first wheats had been baled and removed, and the sites ploughed. These second wheats, in 2001 and 2002, were also of cv. Hereward, and were both sown on 23 September, at 250 seeds m^−2^ and at 300 seeds m^−2^ in Experiments 1 and 2, respectively. Nitrogen was applied to each of the test crops of winter wheat at uniform rates; no adjustments were made to take account of possible differences in the amounts of nitrogen that remained in the soil after the different treatment crops.

Soil samples, used in bioassays to estimate relative amounts of inoculum of the take-all fungus, were taken from all plots after the break crops had been harvested, and also after the first test crops of winter wheat had been harvested. Diseases on the roots and stem bases were assessed on plants sampled from all plots of both of the winter wheat test crops in both spring and summer.

### Experiments 3 and 4

These two experiments were grown alongside, respectively, Experiments 1 and 2 (i.e. in the same fields and over the same 3-year periods). They tested the effects of winter wheat ‘volunteers’ that were present in crops of OSR on the development of take-all in two subsequent crops of winter wheat. Wheat, to simulate volunteers, was either not sown (‘none’) or sown, and then the wheat seedlings destroyed early or late, or not destroyed. These four treatments were tested in combination with two rates of nitrogen (standard practice vs less than standard practice), applied to the OSR, in four complete randomised blocks giving a total of 32 plots in each experiment.

After the second of the two preceding winter wheat crops had been harvested, and the straw baled and removed, the sites of the experiments were ploughed (to minimise numbers of naturally occurring volunteers) before drilling OSR, cv. Apex, at 120 seeds m^−2^ in plots that were 10 m long by 3 m wide, on 28 August 1998 (Experiment 3) or 1 September 1999 (Experiment 4). On the same dates, seed of winter wheat was drilled in Experiment 3 (cv. Abbot at 60 seeds m^−2^) or broadcast in Experiment 4 (cv. Malacca at 90 seeds m^−2^). Ploughing did not completely prevent the growth of naturally occurring volunteers in Experiment 3 so herbicide sprays (cycloxydim at 140 g in 220 L water ha^−1^) to control wheat seedlings were applied to the ‘none’ treatment on 28 September. Similar sprays were applied to the ‘early’ treatment on 4 November (cycloxydim at 150 g in 220 L water ha^−1^), and then to all treatments except ‘not destroyed’ on 8 February and 17 March (cycloxydim at 200 and 450 g, respectively, in 220 L water ha^−1^). In Experiment 4, where naturally occurring volunteers were not a problem, sprays (propaquizafop at 100 g in 220 L water ha^−1^) to control wheat seedlings were applied to the ‘early’ treatment on 4 November and to the ‘late’ treatment on 5 March. Nitrogen was applied to the whole of each experiment in autumn (40 kg ha^−1^ on 25 September and 30 kg ha^−1^ on 19 October, respectively) and in early spring (100 kg ha^−1^ on 10 February and 14 February, respectively). Plots testing standard practice for nitrogen received further applications of 100 kg ha^−1^ on 23 March and 15 March, respectively. Applications of other crop protection chemicals were those considered appropriate for crops of OSR on the Rothamsted farm.

After the OSR had been harvested, the straw was baled and removed, and the sites ploughed, before sowing the first test crops of winter wheat (cv. Hereward) at the same rates and on the same dates as the first test crops in Experiments 1 and 2 (380 seeds m^−2^ on 17 September and 250 seeds m^−2^ on 21 September, respectively). After these had been harvested, the straw baled and removed, and the sites ploughed, second test crops of winter wheat (cv. Hereward) were similarly sown at the same rates and on the same dates as the second test crops in Experiments 1 and 2 (250 seeds m^−2^ on 23 September and 300 seeds m^−2^ on 23 September, respectively). Nitrogen was applied to each of the test crops of wheat at uniform rates; no adjustments were made to take account of possible differences in the amounts of nitrogen that remained in the soil after applying different amounts of nitrogen to the OSR.

Soil samples, used in bioassays to estimate relative amounts of take-all inoculum, were taken from all plots after the OSR, and also after the first test crops of winter wheat had been harvested. Diseases on the roots and stem bases were assessed on plants sampled from all plots of both of the winter wheat test crops in both spring and summer.

### Experiments 5, 6 and 7

These experiments were designed to compare different regimes for managing set-aside/conservation covers established by natural regeneration after crops of winter wheat had been harvested in 1993, 1994 and 1995, respectively. The crops that preceded these winter wheat crops were, respectively, winter wheat, winter beans and spring linseed. Each of the experiments compared five management regimes in which plots were ploughed either early (late May or early June; two regimes) or later (late July or August; three regimes). Re-growth in the early-ploughed plots was either controlled by tine-cultivations during the summer or allowed to develop and then destroyed by spraying with glyphosate before the following test crops were sown (plough/cultivate and plough/glyphosate treatments, respectively). Covers in the later-ploughed plots were either tine-cultivated in May or early June (and a second time, in early July, in Experiment 6), sprayed with glyphosate in May or left to grow until they were ploughed (cultivate/plough, glyphosate/plough and none/plough treatments, respectively). Details of the treatments differed depending on the circumstances in the individual experiments, and covers were topped as necessary to stop plants (especially grass weeds) from shedding seeds, and to facilitate the burial of debris when ploughing. The experiments had multiple objectives, and, in the first year after set-aside, whole plots testing each of the five management regimes were used to compare winter wheat and winter OSR in 4 (Experiment 5) or 3 (Experiments 6 and 7) complete randomised blocks. Nitrogen was applied at standard rates in spring to half plots of the wheat and rape. It was withheld from the other half plots in order to measure the effects of the management regimes on amounts of available N in the soil but these data are not reported here. Similarly, because this article is concerned only with effects on take-all, data from plots testing OSR in the first year after set-aside have been excluded. In the second year after set-aside, all plots were sown with winter wheat, to which N was applied uniformly, but results from plots after OSR are not reported because they had little take-all.

Straw of the wheat crops that preceded Experiments 5, 6 and 7 was chopped and spread. Whole plots, each measuring 26 m long by 12 m wide, were ploughed on, respectively, 3 June or 12 August 1994; 25 May or 22 August 1995; and 15 May or 30 July 1996. Early-ploughed plots were cultivated, using heavy spring-tines, on 6 July and 1 August; 3 July; and 27 June and 30 July, respectively. Alternatively, glyphosate sprays were applied to early-ploughed plots in Experiments 5 and 7 on, respectively, 27 September (wheat plots only) and 15 August; treatment sprays were not considered necessary in Experiment 6 (but see below). Later-ploughed plots in the three experiments were cultivated, using heavy spring-tines, on 3 June; 26 May and 3 July; and 15 May, respectively, or were sprayed with glyphosate on 24 May, 16 May and 30 May. In Experiment 6, couch grass was widespread and, to minimise its potential effects on the following test crops, glyphosate was applied to all plots before ploughing; on 16 May to early-ploughed plots and on 31 July to later-ploughed plots. Glyphosate sprays were applied at rates that ranged from 320 g to 1.44 kg in 200 to 392 L water ha^−1^, depending on the amount and composition of the target covers.

After cultivating the plots to prepare seed beds, the first test crops of winter wheat (cv. Genesis at 380 seeds m^−2^) were sown on 28 September, 26 September and 3 October in Experiments 5, 6 and 7, respectively. Nitrogen, which was tested on sub-plots, each 10 m long, was either not applied or applied at 40 kg ha^−1^ on 13 March, 8 March and 10 March, respectively in Experiments 5, 6 and 7, followed by a further 120 kg ha^−1^ on, respectively, 12 April, 11 April and 4 April. The straw of the first test crops (wheat and OSR) was chopped and spread before the sites were ploughed and all plots sown with winter wheat (cv. Genesis at 380 seeds m^−2^) on 30 September, 16 October and 25 September, respectively, in Experiments 5, 6 and 7.

The distributions of wheat volunteers and other plant species in the set-aside/conservation covers were assessed in late April or early May (before the plots were cultivated or sprayed) by making three quadrat counts per plot. The quadrats measured 1 m × 1 m and each was sub-divided (by strings) into 100 squares each measuring 10 cm × 10 cm. The presence or absence of individual species in each of the small squares was recorded, and the data expressed as frequencies of occurrence (i.e. the mean percentage of squares in which each species was present). In this article we only comment on wheat volunteers and other hosts of the take-all fungus.

Take-all was assessed on wheat volunteers collected from the set-aside/conservation covers in spring. In Experiment 7 only, soil samples were collected from all plots (including those to be sown with OSR) at the end of the set-aside period (in early September; before the first test crops were sown) to investigate effects of the different management regimes on relative amounts of inoculum of the take-all fungus. In the first year after set-aside, plant samples to assess diseases were collected from all plots and sub-plots of wheat in both spring and summer. Wheat in the following year (sown in all plots) was sampled only in summer, when diseases (and yield) were separately assessed in the sub-plots that had been used to test nitrogen in the first test crops.

### Experiments 8 and 9

These experiments were designed to study the effects on take-all of different populations of cereal volunteers in set-aside/conservation covers sown with mustard, and to investigate whether there is, perhaps, a threshold population below which effects on take-all in the following crop(s) are so small that they can be ignored. The experiments also, like experiments 5, 6 and 7, tested the effects of destroying the covers, by ploughing, either ‘early’ or ‘later’. Differently treated rye-grass covers were also tested in these experiments but the results have already been reported elsewhere (Gutteridge *et al*., 2007). The standard errors and degrees of freedom quoted in this article are, however, derived from analyses that included data from the rye-grass plots as well as those sown with wheat/mustard. The target populations of wheat in the wheat/mustard plots could only reliably be obtained by sowing wheat seed (i.e. to simulate volunteers) but it was hoped that this would also result in much less uncontrolled variation, in covers and data, than in the preceding three experiments, which tested covers obtained by natural regeneration. Effects on take-all were measured in three consecutive test crops of winter wheat that followed the different covers.

The two experiments were established after crops of winter wheat had been harvested in 1994 and 1995, respectively. The crops that preceded the wheat were, respectively, OSR and field beans. The wheat stubbles were ploughed, to minimise numbers of naturally occurring wheat volunteers, and then cultivated before sowing winter wheat (cv. Soissons) and mustard (cv. Tilney) in plots that were 10 m long and 6 m wide. The wheat was sown at 0, 4, 9, 50, 200 or 400 seeds m^−2^, thus representing the range of plant populations that might be expected in break crops following wheat (typically towards the bottom end of the range) through to those in set-aside covers, where the growth of volunteers is encouraged and, at the top end of the range, in places where harvested grain was spilt. The mustard was sown at 300 seeds m^−2^ in plots without wheat, at 30 seeds m^−2^ in plots testing the largest rate of wheat, and at 100 seeds m^−2^ in all other plots. The mustard and the larger rates of wheat (50 seeds m^−2^ and above) were drilled on 13 September in Experiment 8, and on 20–21 September in Experiment 9. Plots testing 4 and 9 wheat seeds m^−2^ were sown by hand, on regular grid patterns, starting immediately after the mustard had been drilled. In Experiment 8, hand-sowing was, however, interrupted by rain, after only two of the four blocks had been performed; the remaining two blocks were sown 9 days later. Predictably, these differences in sowing date resulted in small differences in growth stages (GS) during the autumn but by spring no obvious differences were apparent. Hand-sowing in Experiment 9 was completed over three days (20–22 September). Two seeds were sown at each point on the grid so the stated seed rates in these plots are nominal. It was, however, expected that, at these small seed rates, gaps would have a larger effect on the data, and on standard errors, than sometimes two, mutually competing, plants growing at the same point. In Experiment 8, all plots were topped on 11 May. The ‘early’ plots were then ploughed on 12 May, and rotary cultivated on 19 July. ‘Later’ plots were topped a second time on 29 June and also just before ploughing on 17 August. In Experiment 9, ‘early’ plots were ploughed on 17 May, rotary cultivated on 5 July, and then topped on 14 August. ‘Later’ plots were topped on 27 June, and then topped and ploughed on 14 August. For convenience, sprays of glyphosate on 15 August, to destroy weeds in the ‘early’ plots, were applied to the whole experiment. There were, in all, 20 treatment combinations in each experiment (six wheat/mustard covers plus four rye-grass covers × two ploughing dates), which were tested in four fully randomised blocks.

After cultivating the plots to prepare seed beds, the first test crops of winter wheat in Experiments 8 and 9 (cv. Hereward at 375 seeds m^−2^) were sown on 10 October and 25 September, respectively. Straw of the first test crops was baled and removed, and the sites ploughed and cultivated, before the second test crops, also of cv. Hereward, were sown, on 9 October (at 325 seeds m^−2^) and 25 September (at 350 seeds m^−2^), respectively. Third test crops were of cv. Rialto, and were sown after the sites had been similarly prepared, on 26 September (at 350 seeds m^−2^) and 13 October (at 380 seeds m^−2^), respectively.

Soil samples, used in bioassays to estimate relative amounts of inoculum of the take-all fungus, were taken at random from across the sites of both experiments at the start of the treatment (i.e. set-aside) year (50 cores), and then from all plots in late April or early May and again in late July (shortly before ‘early’ and ‘later’ ploughing, respectively). Take-all was assessed on wheat plants sampled from the set-aside covers in spring and summer but only from plots sown at 50 seeds m^−2^ or above. Diseases were assessed on plants sampled from the first and second winter wheat test crops in both spring and summer but the third wheats were only sampled in summer.

### Experiments 10 and 11

These relatively simple experiments were intended to provide preliminary information on, first, the effects of interrupting with set-aside/conservation covers a sequence of winter wheat crops in which severe take-all had occurred, and in which TAD was expected to have established (Experiment 10), and, second, to explore the possibility of substituting set-aside/conservation covers for winter wheat during the build-up phase of a take-all epidemic in order to establish effective TAD while avoiding the worst effects of take-all disease on crop yield (Experiment 11).

Both experiments were started in 2001, and compared three treatments. Experiment 10 was sown in a field that had previously grown four consecutive crops of winter wheat, the immediately preceding one of which (i.e. the fourth wheat, harvested in 2000) had very severe take-all. It was, therefore, expected that TAD would be evident in winter wheat grown in 2001. In contrast, Experiment 11 followed a second crop of winter wheat, in which take-all was relatively slight so the disease was expected to be much more severe in the subsequent crop(s). In both experiments, the treatments were tested in four fully randomised blocks in plots that were 10 m long by 6 m wide. In Experiment 10, the treatments (in 2001) were winter wheat or set-aside/conservation covers that were either topped as necessary or sprayed with glyphosate as early as allowed by conditions and the prevailing regulations. Test crops of winter wheat were grown in the following 2 years. The treatments tested in Experiment 11 were winter wheat (in 2001–2003), set-aside/conservation covers in 2001 (followed by winter wheat in 2002 and 2003) and set-aside/conservation covers in 2001 and 2002 (followed by winter wheat in 2003). The covers in this experiment were all topped.

Wheat in Experiment 10 (cv. Hereward, in 2001–2003) was sown on 23 September (at 250 seeds m^−2^), 22 September (at 300 seeds m^−2^) and 25 September (at 350 seeds m^−2^), respectively. Set-aside/conservation covers (in 2001) were a mixture of wheat and OSR (cv. Malacca at 150 seeds m^−2^ and cv. Apex at 20 seeds m^−2^, respectively), both sown on 5 September. The covers were either sprayed with glyphosate (on 11 June) or topped (on 21 and 28 June). The site of the experiment was ploughed on 18 August 2000, before sowing the treatment crops and covers, and again on 5 September 2001 and 17 September 2002, before sowing the subsequent test crops (after straw of the preceding crops or covers had been chopped and spread). Wheat in Experiment 11 (cv. Hereward, in 2001–2003) was sown on 23 September (at 250 seeds m^−2^), 22 September (at 300 seeds m^−2^) and 17 September (at 250 seeds m^−2^), respectively. Set-aside/conservation covers in 2001 were sown on 5 September (wheat cv. Malacca at 150 seeds m^−2^ with OSR cv. Apex at 20 seeds m^−2^), and in 2002 on 10 September (wheat cv. Hereward at 100 seeds m^−2^ with OSR cv. Apex at 40 seeds m^−2^). They were topped on 21 June in 2001 but not topped in 2002. The successive treatment and test crops in Experiment 11 were sown after the site had been ploughed on 1, 6 and 5 September, respectively (and after straw of the preceding crops or covers had been chopped and spread).

Diseases were assessed on wheat plants sampled from the wheat crops and from the set-aside/conservation covers in both spring and summer. Soil samples were taken from all plots in both experiments in late August 2001 to assess, in bioassays, effects on take-all inoculum of the first year's treatments.

### Soils and husbandry

Experiments 1–4, and 10 and 11, were on the Rothamsted Farm, on soils that are mostly flinty silty clay loams over clay-with-flints (Batcombe and related series). Experiments 5–7 were on the adjoining Scout Farm (managed with the Rothamsted Farm as a single unit), on soils that are mostly deep flinty loams with loamy, gravelly or clayey substrata (Charity complex). Experiments 8 and 9 were on the Woburn Experimental Farm, in Bedfordshire, on soils that are mostly sandy loams or sandy clay loams overlying Lower Greensand (Lowlands and related series) or Chalky Boulder Clay (Beccles).

Treatments applied to experimental crops and covers are described above. Subsequent test crops were, in most respects, grown according to standard practice on the Rothamsted and Woburn farms. Nitrogen fertiliser was applied as split dressings in spring, usually in March (sometimes in February) and in April (sometimes in May) at rates that ranged from 40 to 60 and from 90 to 160 (but usually 130–160) kg ha^−1^, respectively). Crops grown on the light sandy soils on the Woburn Farm often display transitory symptoms of, especially, manganese deficiency in spring so all test crops in Experiments 8 and 9 were sprayed with products containing this, and sometimes other trace elements. Applications of crop protection chemicals included herbicide and fungicide sprays but the latter did not include azoxystrobin, which has been shown to have activity against the take-all fungus (Jenkyn *et al*., 2000). In some experiments, fungicides with recognised activity against the eyespot pathogens (*Oculimacula yallundae* and *Oculimacula acuformis*) were also avoided so that effects of the treatments on eyespot disease could also be studied. Grain yields were measured after using a combine harvester to take a cut through the centre of each plot or sub-plot, and were adjusted to 85% dry matter. Thousand-grain weights were determined after the grain had been dried. More detailed information on husbandry for most of the crops can be obtained from the ‘Yields of the Field Experiments’ for the appropriate years, published by Rothamsted Experimental Station or, latterly, IACR-Rothamsted (not produced after 2000).

### Disease assessments

All winter wheat test crops were sampled in summer to assess take-all on the roots, and diseases affecting the stem bases. First wheats, and second wheats except those in Experiments 5, 6 and 7, were also sampled in spring, principally to assess take-all. Summer samples, which were taken in June/July at GS (Zadoks *et al*., 1974) 69–77, consisted of 10, 20-cm lengths of row per plot except those from third wheats in Experiments 8 and 9, and from wheats in the first 2 years of Experiments 10 and 11, which consisted of 5, 20-cm lengths of row per plot. Spring samples were taken in April/early May (GS 22–33), and consisted of 5 × 15 or 20 cm of row per plot.

The sampled plants were washed and the root systems of spring samples examined, as soon as possible, under water in a white dish. Total numbers of plants in each sample, and numbers of healthy and take-all affected root axes on each plant were counted. Numbers of plants and shoots affected by stem-base diseases were also counted. Samples taken in summer were washed and dried, and then stored for assessment at a later date. The dried root systems were soaked in water, and then, as in spring, examined under water in a white dish. Take-all on individual root systems was usually assessed on a 0–5 scale as follows: absent (0); slight, affecting 1–10% of the root system (1); slight, affecting 11–25% of the root system (2); moderate, affecting 26–50% of the root system (3); moderate, affecting 51–75% of the root system (4) and severe, affecting more than 75% of the root system (5). A take-all index (TAI) on a 0–100 scale was calculated by adding the percentage of plants in each category multiplied by its score value and dividing the total by 5. The 0–5 scale was adopted part-way through these experiments so indices for a few of the earlier test crops are based on a 0–3 scale, in which the slight and moderate categories described above were not sub-divided. Summer samples were also used to determine percentages of straws with slight, moderate or severe eyespot (Scott & Hollins, 1974) and with sharp eyespot (*Rhizoctonia cerealis*) and brown foot rot (*Fusarium* spp. and *Microdochium* spp.).

As this article is principally concerned with take-all, data for diseases affecting the stem bases are not reported in detail unless they were unusually common or there was evidence of significant and consistent differences between treatments.

### Soil infectivity bioassays

Relative amounts of take-all inoculum in the soil were estimated using bioassays (Gutteridge & Hornby, 2003). Usually, five soil cores (each 5 cm diameter × *c*. 10 cm deep) were taken at random from each plot but 10 cores were taken from each plot in Experiment 7. Fifty cores were taken for the pre-treatment assessments of the sites used for Experiments 8 and 9. Each soil core was placed, inverted, into an 11 cm tall white plastic beaker (drinking cups; manufactured by Mono Containers Ltd, ref. PV11, and supplied by Sarrett Office Supplies Ltd, Herts, UK) previously drilled with four 3 mm diameter drainage holes and containing 50 mL of coarse sand. Ten germinated wheat seeds were placed on the soil and covered with horticultural grit. The beakers were placed, randomised, in a controlled environment room (16-h day, radiation 300 µE m^−2^ s^−1^, 70% RH, day/night temperatures 15/10°C), with water applied twice weekly. After 5 weeks the plants were removed, their roots washed, and the presence or absence of take-all lesions recorded on each main root axis. The mean percentage of roots infected in each soil core was calculated as a measure of soil infectivity.

### Statistical analyses

Differences between treatments were tested by analyses of variance using Genstat. References to significant differences in the text can be assumed to be at *P* < 0.05 unless otherwise stated. For the analysis of disease data expressed as percentages, a mean value (*P*%) was calculated for each plot and a logit transformation used (i.e. 0.5ln[(*P*% + 0.05)/(100.05% − *P*%)]), a formula that avoids problems arising from *P*% values of 0 or 100%. Standard errors apply to the quoted logit means, and the quoted percentage values were obtained by back transformation.

## Results

### Experiments 1 and 2

Among the break crops, peas failed in Experiment 1, and lupins grew much better in that experiment than they did in Experiment 2. Despite efforts to control them, there were some patchily distributed wheat volunteers in Experiment 1 but numbers were mostly small except in one plot of lupins. There were fewer wheat volunteers in Experiment 2 but OSR volunteers (presumably from the crop grown 3 years before) did occur, especially in two of the bean plots. The plots of winter wheat in the break-crop years, which were third consecutive wheats, had significant take-all but it was more severe in Experiment 1 (TAI = 76) than in Experiment 2 (TAI = 51).

Amounts of take-all inoculum in the soil after the break crops had been harvested in Experiment 1 were very small, and not significantly different (Table 1). There were similarly small amounts of inoculum in the soil in Experiment 2 but significantly more after linseed than after the other break crops (Table 2); this was not, however, reflected in the amounts of take-all in the first wheat. There was most inoculum after winter wheat, and especially in Experiment 1, apparently reflecting differences in the severity of the disease in the preceding crops. Take-all in the first wheat crops after the breaks was generally more prevalent in Experiment 1 than in Experiment 2, especially in summer, but it remained slight in both, and differences between the break crops were mostly small and not significant. There was much more take-all in wheat after wheat (i.e. fourth wheats) but less than there had been in the preceding (third) wheats; it was moderately severe in Experiment 1, and only slight in Experiment 2. Grain yields in Experiment 1 were relatively small but wheat after oats yielded significantly more than wheat after the other break crops (7.84 and 6.64 t ha^−1^, respectively). The poor yields are, however, more likely to have been a consequence of severe lodging than of take-all, and this may also explain the apparent benefit of oats. Plot areas affected by lodging in July averaged about 20% in wheat after oats and about 75% in wheat after the other breaks. In contrast, the very much poorer yield of wheat after wheat (3.81 t ha^−1^) was probably due in large part to the effects of take-all because the area lodged in these plots was only about 5%. The differences in yield were at least partly a consequence of differences in thousand-grain weights, which averaged 36.1, 31.9 and 26.6 g after oats, non-graminaceous breaks and wheat, respectively. Yields were much larger in Experiment 2, averaging 8.89 t ha^−1^ from wheats grown after a break but, despite relatively slight take-all, were about a tonne per hectare less from wheat after wheat (7.74 t ha^−1^).

**Table 1:** Take-all infectivity of soil, measured in a bioassay, after harvesting different test crops, and amounts of take-all and grain yield measured in the following, first, test crops of winter wheat (Experiment 1)

Treatment Crop(in 1999)	Infectivity of Soil(% roots)^a^	Take-All in Spring		Take-All in Summer		Grain Yield(t ha^−1^)
		Total (% plants)	No. Roots (plant^−1^)	Total (% plants)	Take-All Index (0–100)	
Oilseed rape	−2.49 (0.2)	−1.12 (9.0)	0.14	−0.94 (12.8)	6.5	6.29
Linseed	−2.58 (0.1)	−1.28 (6.7)	0.13	−1.21 (7.7)	8.2	7.15
Lupins	−2.63 (0.0)	−1.27 (6.8)	0.17	−0.54 (24.7)	11.7	6.75
Beans	−2.10 (1.0)	−1.27 (6.8)	0.10	−0.79 (16.7)	10.4	6.47
Peas	−2.72 (0.0)	−1.29 (6.5)	0.32	−0.56 (24.3)	13.5	6.53
Oats	−2.18 (0.8)	−0.75 (17.7)	0.33	−0.58 (23.2)	10.4	7.84
Wheat	+0.66 (78.5)	+0.63 (77.4)	3.57	+0.74 (80.8)	57.7	3.81
SED (18 d.f.)	0.385	0.399	0.258	0.281	5.21	0.325

a Data expressed as percentages were transformed before analysis and are shown as logit means with, in parentheses, the corresponding percentage values obtained by back transformation.

**Table 2:** Take-all infectivity of soil, measured in a bioassay, after harvesting different test crops, and amounts of take-all and grain yield measured in the following, first, test crops of winter wheat (Experiment 2)

Treatment Crop (in 2000)	Infectivity of Soil (% roots)^a^	Take-All in Spring		Take-All in Summer		Grain Yield (t ha^−1^)
		Total (% plants)	No. Roots (plant^−1^)	Total (% plants)	Take-All Index (0–100)	
Oilseed rape	−2.88 (0.0)	−1.56 (3.7)	0.05	−1.65 (3.1)	0.8	9.00
Linseed	−1.64 (3.1)	−1.27 (6.8)	0.08	−1.97 (1.4)	0.3	9.08
Lupins	−2.27 (0.6)	−0.81 (16.2)	0.32	−1.23 (7.4)	1.9	8.61
Beans	−2.81 (0.0)	−1.54 (3.9)	0.05	−1.65 (3.1)	0.6	9.61
Peas	−2.67 (0.0)	−1.48 (4.4)	0.07	−1.84 (2.0)	0.5	8.47
Oats	−2.75 (0.0)	−1.36 (5.6)	0.13	−1.49 (4.4)	1.4	8.57
Wheat	−0.09 (44.8)	+0.64 (77.8)	1.81	+0.18 (58.5)	18.1	7.74
SED (18 d.f.)	0.314	0.432	0.292	0.309	0.90	0.631

a Data expressed as percentages were transformed before analysis and are shown as logit means with, in parentheses, the corresponding percentage values obtained by back transformation.

Predictably, there was much more take-all inoculum in the soil after the first test crop of wheat than there had been after the break crops, especially in Experiment 1 where take-all in the first wheat was most severe. Despite this, take-all in the following, second, test crop of winter wheat became more severe in Experiment 2 than in Experiment 1. In the latter experiment, there was significantly less inoculum where the wheat followed wheat (i.e. after four consecutive crops of wheat) than where the wheat followed a break, and that was reflected in the amounts of take-all that developed in the following, second, test crop of winter wheat (Table 3). There were, however, no differences between the break crops in either amounts of take-all inoculum in the soil or in symptoms of the disease. In Experiment 2, there were no clear differences between the test crops in the amounts of take-all inoculum in the soil, even after four consecutive crops of wheat (Table 4). Incidence and severity of take-all in the second test crops were, however, consistently, and significantly, smaller where oats had been grown 2 years before than where the other break crops had been grown. The disease also tended to be less severe in the fifth consecutive wheat crop than in the second wheat crops after non-graminaceous breaks. Grain yields obtained from the second test crops reflected the differences in severity of take-all that apparently resulted from growing different crops 2 years before. In Experiment 1, the average yield of all second wheat crops after break crops was 2.67 t ha^−1^ less than the yield of the fifth consecutive wheat (4.14 and 6.81 t ha^−1^, respectively). On average, the second wheat crops in Experiment 2 gave over two tonnes per hectare more grain after oats than they did after the non-graminaceous break crops (6.74 and 4.54 t ha^−1^, respectively) while the fifth consecutive wheat gave an intermediate yield of 5.52 t ha^−1^. However, while the relatively larger yield of grain from the second wheats after oats was probably due to the associated lesser severity of take-all, there is some doubt as to whether the difference between oats and the other break crops was a real effect. Data from the plots after oats were very variable, and the observed decrease in mean severity of take-all and increase in mean grain yield, relative to the other break crops, was largely attributable to results from just one of the four replicate plots, which had very large residuals.

**Table 3:** Take-all infectivity of soil, measured in a bioassay, after harvesting first test crops of winter wheat in Experiment 1, and amounts of take-all and grain yield measured in the following, second, test crops of winter wheat

Treatment Crop (in 1999)	Infectivity of Soil (% roots)^a^	Take-All in Spring		Take-All in Summer		Grain Yield (t ha^−1^)
		Total (% plants)	No. Roots (plant^−1^)	Total (% plants)	Take-All Index (0–100)	
Oilseed rape	+0.18 (58.2)	+1.88 (97.2)	4.7	+2.30 (98.5)	79.3	3.85
Linseed	+0.18 (58.5)	+2.01 (97.7)	4.4	+2.04 (97.8)	76.4	4.27
Lupins	+0.36 (66.6)	+1.94 (97.5)	4.4	+2.41 (98.7)	78.8	3.75
Beans	+0.12 (55.5)	+2.07 (97.9)	5.3	+2.25 (98.4)	79.3	3.81
Peas	+0.19 (58.7)	+1.60 (95.6)	4.5	+2.31 (98.5)	76.3	4.67
Oats	+0.39 (68.2)	+1.67 (96.1)	3.8	+2.30 (98.5)	80.4	4.50
Wheat	-0.13 (43.1)	+0.77 (81.9)	1.9	+0.99 (87.3)	56.9	6.81
SED (18 d.f.)	0.142	0.299	0.82	0.248	6.72	0.383

a Data expressed as percentages were transformed before analysis and are shown as logit means with, in parentheses, the corresponding percentage values obtained by back transformation.

**Table 4:** Take-all infectivity of soil, measured in a bioassay, after harvesting first test crops of winter wheat in Experiment 2, and amounts of take-all and grain yield measured in the following, second, test crops of winter wheat

Treatment Crop (in 2000)	Infectivity of Soil (% roots)^a^	Take-All in Spring		Take-All in Summer		Grain Yield (t ha^−1^)
		Total (% plants)	No. Roots (plant^−1^)	Total (% plants)	Take-All Index (0–100)	
Oilseed rape	−0.43 (29.4)	+1.52 (94.9)	6.6	+2.55 (98.9)	94.7	5.07
Linseed	−0.28 (35.8)	+1.66 (96.0)	7.2	+2.58 (98.9)	94.8	4.36
Lupins	−0.08 (45.8)	+1.65 (95.9)	5.9	+2.58 (98.9)	90.0	4.38
Beans	−0.12 (43.4)	+1.63 (95.8)	5.6	+2.55 (98.9)	91.6	4.59
Peas	−0.18 (40.5)	+1.47 (94.5)	6.2	+2.55 (98.9)	92.5	4.29
Oats	−0.20 (39.9)	+0.52 (73.3)	3.7	+1.86 (97.1)	77.7	6.74
Wheat	−0.29 (35.6)	+1.38 (93.6)	4.2	+2.59 (98.9)	80.0	5.52
SED (18 d.f.)	0.147	0.349	1.23	0.242	6.78	0.955

a Data expressed as percentages were transformed before analysis and are shown as logit means with, in parentheses, the corresponding percentage values obtained by back transformation.

There was little evidence of significant or consistent effects of the different treatment crops on the incidence or severity of stem base diseases, or on the incidence of gout fly (*Chlorops pumilionis*) in spring, in the following crops of wheat.

### Experiments 3 and 4

Amounts of inoculum in the soil after the OSR had been harvested, measured in bioassays, were relatively small in both experiments but greater in Experiment 3 than in Experiment 4 (Tables 5 and 6). In Experiment 4, but not in Experiment 3, there was significantly more inoculum after OSR given the smaller amount of nitrogen than after that given the standard amount (0.9 vs 0.1% roots infected). In both experiments, there was more inoculum where volunteers had not been destroyed than where they were not present or were destroyed ‘early’ but only in Experiment 4 were these differences significant. There tended to be intermediate amounts of inoculum where volunteers were destroyed ‘late’. Because differences between the volunteer treatments were qualitative, not quantitative, there were few options for more detailed analyses of the data. It was, however, anticipated that survival and multiplication of inoculum of Ggt would increase progressively in plots testing ‘no’ volunteers, volunteers destroyed ‘early’, volunteers destroyed ‘late’ and volunteers not destroyed. In further analyses of the bioassay data from Experiment 3, in which the *F*-test in the analyses of variance detected no significant differences between the volunteer treatments, the anticipated risk generated by these treatments was arbitrarily rated from 1 to 4, respectively. These analyses provided evidence that measured amounts of inoculum increased with anticipated risk in an approximately linear fashion, which, for logit transforms of per cent roots infected, was significant (*P* = 0.04).

**Table 5:** Take-all infectivity of soil, measured in a bioassay, after harvesting crops of oilseed rape, grown with or without winter wheat to simulate volunteers, and amounts of take-all and grain yield measured in the following, first, test crops of winter wheat (Experiment 3)

Wheat in OSR (in 1999)	Infectivity of Soil (% roots)^a^	Take-All in Spring		Take-All in Summer		Take-All Index (0–100)	Grain Yield (t ha^−1^)
		Total(% plants)	No. Roots (plant^−1^)	Total (% plants)	Mod. + Sev. (% plants)		
None	−2.10 (1.0)	−1.20 (7.8)	0.16	−0.97 (12.1)	−1.76 (2.4)	5.8	7.19
Destroyed early	−2.13 (0.9)	−1.22 (7.5)	0.11	−0.51 (26.2)	−1.25 (7.1)	14.2	6.93
Destroyed late	−1.78 (2.3)	−0.88 (14.2)	0.40	−0.33 (33.4)	−1.01 (11.2)	16.1	6.49
Not destroyed	−1.53 (4.0)	−0.35 (32.9)	0.89	+0.24 (61.0)	−0.53 (25.2)	32.1	6.60
SED (21 d.f.)	0.288	0.238	0.174	0.212	0.288	4.87	0.267

a Data expressed as percentages were transformed before analysis and are shown as logit means with, in parentheses, the corresponding percentage values obtained by back transformation.

**Table 6:** Take-all infectivity of soil, measured in a bioassay, after harvesting crops of oilseed rape, grown with or without winter wheat to simulate volunteers, and amounts of take-all and grain yield measured in the following, first, test crops of winter wheat (Experiment 4)

Wheat in OSR (in 2000)	Infectivity of Soil (% roots)^a^	Take-All in Spring		Take-All in Summer		Take-All Index (0–100)	Grain Yield (t ha^−1^)
		Total(% plants)	No. Roots(plant^−1^)	Total (% plants)	Mod. (% plants)^b^		
None	−2.55 (0.1)	−1.56 (3.8)	0.07	−1.73 (2.5)	−2.44 (0.3)	1.0	8.20
Destroyed early	−2.78 (0.0)	−1.81 (2.1)	0.06	−1.75 (2.4)	−2.24 (0.6)	1.7	8.56
Destroyed late	−2.03 (1.2)	−1.61 (3.4)	0.06	−1.81 (2.1)	−2.45 (0.2)	0.7	8.31
Not destroyed	−1.92 (1.6)	−1.12 (9.1)	0.25	−1.37 (5.5)	−2.09 (1.0)	3.5	8.46
SED (21 d.f.)	0.274	0.231	0.068	0.263	0.148	1.36	0.286

a Data expressed as percentages were transformed before analysis and are shown as logit means with, in parentheses, the corresponding percentage values obtained by back transformation.

b There were no plants with severe symptoms.

There was more take-all in the first test crops of winter wheat after OSR in Experiment 3 than in Experiment 4 but, in both experiments, symptoms were mostly slight. There was, though, consistently most disease where volunteers in the OSR had not been destroyed, reflecting differences in the amounts of inoculum detected in the bioassays. In Experiment 3, there were no significant differences in spring between plots testing ‘no’ volunteers and volunteers destroyed ‘early’, which reflected the similar amounts of inoculum detected in the bioassay. In summer, however, there was less take-all where there were ‘no’ volunteers than where they were present, and it increased progressively as their destruction was delayed or neglected. Applying different amounts of N to the OSR had no significant effects on the disease (not shown). In Experiment 4, the differences between volunteer treatments in spring were significant, almost entirely reflecting the larger amounts of take-all in plots where volunteers in the OSR had not been destroyed than in plots testing the other treatments; differences between the other treatments were very small and not significant. In summer, differences between the volunteer treatments, judged by the *F*-test in the analysis of variance, were not significant although there was, again, most take-all where volunteers had not been destroyed. There was, however, evidence of a significant interaction between the volunteer and nitrogen treatments (especially for per cent plants with moderate symptoms; *P* = 0.02). This interaction was a consequence of more severe take-all in plots where volunteers in the OSR had not been destroyed and N had been applied at less than the standard rate than in plots testing all other treatment combinations (12.0 vs 2.3% plants with take-all; 2.7 vs 0.3% with moderate symptoms). Wheat in Experiment 3 that followed OSR that contained ‘no’ volunteers gave the largest yield of grain (7.19 t ha^−1^); about 10% more than the average for wheat after OSR in which volunteers were destroyed ‘late’ or were not destroyed. Grain yields in Experiment 4, which averaged 8.38 t ha^−1^, were larger than in Experiment 3, and differences between the treatments were small and not significant, reflecting the very small amounts of take-all in these crops.

Results of bioassays of soil sampled after the first crops of wheat following OSR had been harvested showed that inoculum of the take-all fungus was abundant (Tables 7 and 8). It appeared to be even more common in Experiment 4, where symptoms of take-all in the first wheat had been very slight, than in Experiment 3, where there was slightly more disease. Take-all in the following, second, crops of winter wheat became severe in both experiments, but especially in Experiment 4, and grain yields were correspondingly small. Differences between the treatments were mostly small and not significant except that in Experiment 3 the proportions of plants with moderate or severe take-all in summer were smaller where volunteers in the OSR had been destroyed ‘late’ or were not destroyed than where there were ‘none’ or they were destroyed ‘early’. These differences in the severity of take-all in Experiment 3 were associated with differences in thousand-grain weights (35.8 and 34.7 g, respectively) but not in total yields of grain.

**Table 7:** Take-all infectivity of soil, measured in a bioassay, after harvesting first test crops of winter wheat in Experiment 3, and amounts of take-all and grain yield measured in the following, second, test crops of winter wheat

Wheat in OSR (in 1999)	Infectivity of Soil (% roots)^a^	Take-All in Spring		Take-All in Summer			Grain Yield (t ha^−1^)
		Total (% plants)	No. Roots (plant ^−1^)	Total (% plants)	Mod. + Sev. (% plants)	Take-All Index (0–100)	
None	+0.29 (63.8)	+1.73 (96.5)	4.5	+2.05 (97.9)	+1.10 (89.5)	75.8	4.71
Destroyed early	+0.02 (50.6)	+1.86 (97.1)	4.2	+2.25 (98.4)	+1.24 (91.8)	79.3	4.87
Destroyed late	0.00 (49.4)	+1.77 (96.7)	4.1	+1.85 (97.1)	+0.55 (74.5)	67.3	5.36
Not destroyed	+0.26 (62.1)	+1.82 (96.9)	4.2	+1.76 (96.6)	+0.50 (72.7)	66.8	4.91
SED (21 d.f.)	0.147	0.185	0.50	0.265	0.284	5.25	0.466

a Data expressed as percentages were transformed before analysis and are shown as logit means with, in parentheses, the corresponding percentage values obtained by back transformation.

**Table 8:** Take-all infectivity of soil, measured in a bioassay, after harvesting first test crops of winter wheat in Experiment 4, and amounts of take-all and grain yield measured in the following, second, test crops of winter wheat

Wheat in OSR (in 2000)	Infectivity of Soil (% roots)^a^	Take-All in Spring		Take-All in Summer			Grain Yield (t ha^−1^)
		Total (% plants)	No. Roots (plant ^−1^)	Total (% plants)^b^	Mod. + Sev. (% plants)	Take-All Index (0–100)	
None	+0.31 (64.5)	+1.73 (96.5)	5.5	(100.0)	+2.01 (97.7)	92.7	4.05
Destroyed early	+0.51 (72.9)	+1.86 (97.1)	6.1	(100.0)	+2.16 (98.2)	95.3	3.44
Destroyed late	+0.52 (73.2)	+1.96 (97.5)	5.8	(100.0)	+2.26 (98.4)	94.0	3.94
Not destroyed	+0.45 (70.4)	+2.01 (97.7)	6.4	(100.0)	+1.94 (97.5)	94.2	3.52
SED (21 d.f.)	0.131	0.248	0.50	—	0.244	2.06	0.439

a Data expressed as percentages were transformed before analysis and are shown as logit means with, in parentheses, the corresponding percentage values obtained by back transformation.

b Data not analysed because all of the sampled plants were affected by take-all.

There were few significant or consistent effects on wheat stem-base diseases but in Experiment 3, brown foot rot in the second wheat was less common where N had been applied to the OSR at the standard rate than where it had been applied at the less than standard rate (17.6 and 25.5% straws affected, respectively). In Experiment 4, sharp eyespot in the first wheat was more common where volunteers in the OSR had not destroyed than in plots where there were none or they had been destroyed (48.7% straws affected vs a mean of 28.0%). The disease was also more common, in both first and second wheat crops, where N had been applied to the OSR at the standard than at the less than standard rate (37.5 and 28.2% straws, respectively, in the first wheat; 17.6 and 8.0% straws, respectively, in the second wheat).

### Experiments 5, 6 and 7

There was much variation in the extent and composition of the covers that developed in these experiments, by natural regeneration, and which were used to test the management regimes. This was especially so in Experiment 5 where in two of the replicate blocks (Blocks 3 and 4) there was much less vegetation, and much more bare ground, than in the other two. These differences were, to an extent, reflected in the frequencies of wheat volunteers. The overall mean, in the plots that were subsequently sown with winter wheat, was 58% but the figures for blocks 1–4 were, respectively, 94, 62, 52 and 22%. There was much variation within blocks, as well as between blocks, and especially in the blocks with least cover. Other graminaceous species, mostly black-grass (*Alopecurus myosuroides*), were relatively uncommon in this experiment but their distribution was also patchy. In Experiment 6, there was much less variation in numbers of wheat volunteers, with mean frequencies in blocks 1–3 of 46, 49 and 44%, respectively, but their distribution was, again, patchy. The covers in this experiment also contained several, very patchily distributed, grass weeds. The commonest was couch grass (*Elytrigia repens*, with a mean frequency of 25%) but others included black-grass (5%), soft brome (*Bromus hordeaceus*, 3%) and perennial rye-grass (*Lolium perenne*, 3%). The mean frequency of wheat volunteers in Experiment 7 was 58% but there were differences between blocks (mean frequencies in blocks 1–3 of 44, 53 and 78%, respectively). The commonest grass weeds in this experiment were couch grass (with a mean frequency of 5%), soft brome (5%) and black-grass (2%); all of them were very patchy in their distribution. Take-all, assessed on wheat volunteers sampled from the set-aside covers in spring, affected 27.3, 22.8 and 33.7% of plants, respectively, in Experiments 5, 6 and 7 with, on average, 0.43, 0.77 and 0.85 infected roots plant^−1^.

Take-all in the first test crops of winter wheat, assessed in summer, was least common in Experiment 6 and most common in Experiment 7, where it became moderately severe in the none/plough treatment (Table 9). In Experiment 5 there was much more take-all in the two blocks where wheat volunteers and other vegetation in the set-aside year were most common than in the two blocks where there was little green cover and much bare ground. Numbers of plants affected by the disease in summer, for example, averaged 36.5 and 9.8%, respectively. Nitrogen consistently decreased the incidence and severity of take-all in the first test crops in summer but not always significantly. Percentages of plants affected by the disease, for example, in sub-plots to which N was or was not applied averaged 14.3 vs 27.4; 6.0 vs 15.4; and 41.6 vs 56.3 in Experiments 5, 6 and 7, respectively (not significant in Experiment 7). In Table 9, which summarises effects of the different management regimes on take-all, only the data from sub-plots to which N was applied are presented because they are agronomically most relevant. There was, however, little evidence in these data of significant or consistent differences between the regimes in either the incidence or severity of take-all. This may reflect, at least in part, the variability in the covers that were used to test the regimes, and especially in Experiment 5. In Experiment 6, there was less take-all in the plots that were ploughed or cultivated early than in those that were sprayed with glyphosate or were only topped before being ploughed late but only in percentages of plants affected in summer were differences significant. In Experiment 7, amounts of take-all inoculum estimated from bioassays of soil sampled at the end of the set-aside period and before the first test crops were sown, tended to be smaller in early-ploughed plots that were subsequently sprayed with glyphosate than in plots testing other treatments (5.0 vs 12.1% roots infected) but the difference was not significant. In the following wheat (Table 9), there was a tendency for the disease to be less common and severe in the early- than in the later-ploughed plots, especially in spring, but the differences were, again, not significant. As expected, yields of grain (not shown) were, in all three experiments, significantly larger where N had been applied than where it had not. Yields associated with the different management regimes differed more in sub-plots where N had not been applied than where it had (despite smaller mean yields in the former) but differences between regimes were neither significant nor consistent.

**Table 9:** Take-all in first test crops of winter wheat grown after set-aside/conservation covers that had been subjected to different management regimes in Experiments 5, 6 and 7

	Management Regime					SED (d.f.)^b^
	Plough Cultivate	Plough Glyphosphate	Cultivate Plough	Glyphosphate Plough	None Plough	
First wheat in spring (% plants affected)^a^						
Experiment 5	−1.61 (3.3)	−0.99 (11.6)	−1.20 (7.8)	−1.79 (2.2)	−1.01 (11.3)	0.363 (24.7)
Experiment 6	−1.34 (6.0)	−2.20 (0.7)	−1.86 (1.9)	−0.95 (12.5)	−1.05 (10.4)	0.436 (18.0)
Experiment 7	−1.24 (7.2)	−1.63 (3.2)	−0.99 (11.7)	−0.91 (13.3)	−0.87 (14.5)	0.465 (14.8)
First wheat in spring (infected roots plant^−1^)						
Experiment 5	0.07	0.19	0.16	0.03	0.31	0.137 (27.0)
Experiment 6	0.11	0.00	0.02	0.42	0.23	0.135 (16.9)
Experiment 7	0.10	0.05	0.26	0.31	0.32	0.289 (13.7)
First wheat in summer (% plants affected)^a^						
Experiment 5	−0.83 (15.6)	−0.61 (22.4)	−0.83 (15.5)	−1.35 (5.8)	−0.76 (17.4)	0.480 (24.3)
Experiment 6	−2.06 (1.1)	−1.68 (2.9)	−1.45 (4.7)	−0.81 (15.9)	−0.68 (20.0)	0.391 (17.9)
Experiment 7	−0.17 (41.1)	−0.62 (21.9)	+0.11 (55.1)	−0.15 (42.1)	+0.03 (50.8)	0.570 (14.7)
First wheat in summer (Take-all Index)						
Experiment 5	11.8	15.5	14.4	4.5	18.3	9.31 (20.3)
Experiment 6	0.6	4.0	1.7	10.3	9.1	5.26 (17.8)
Experiment 7	28.1	18.7	32.8	28.6	41.4	15.44 (14.7)

a Data expressed as percentages were transformed before analysis and are shown as logit means with, in parentheses, the corresponding percentage values obtained by back transformation.

b Degrees of freedom differ, within the same experiment, because they have been weighted to take account of differences in the residual mean squares in the different strata, in the analyses of variance, from which the quoted standard errors were derived.

Mean incidence of take-all in the second test crops of winter wheat in summer (assessed in the sub-plots to which N had been applied in the previous year) was greater than incidence in the first wheat crops in Experiment 5 (23.4 vs 14.3% plants affected) and, especially, in Experiment 7 (98.7 vs 41.6%) but was very similar in Experiment 6 (6.5 vs 6.0%). There was no evidence that the different management regimes had any effect on take-all in the second test crops of winter wheat but in Experiment 5 there was, again, more take-all in the two blocks that had had the most green cover in the set-aside year than in the two blocks that had little cover (respectively 63.4 and 22.2% plants affected, e.g.). Incidence and severity of the disease were also significantly smaller in Experiment 5 where N had been applied to the first wheat than where it had not (23.4 and 61.8% plants affected; TAIs of 19.0 and 31.8, respectively) but there was no evidence of similar residual effects of N in Experiments 6 or 7. Grain yields of the second test crops of winter wheat were not significantly or consistently affected by either management regimes or by the application of N to the first test crops of wheat.

The commonest diseases affecting the stem bases of the first winter wheat test crops in summer were eyespot and brown foot rot, both of which were significantly affected by N. Incidence of eyespot was relatively unaffected but the disease was consistently less severe where N was applied than where it was not (mean percentages of straws with moderate or severe symptoms in Experiments 5, 6 and 7 were, respectively 11.5 vs 18.7; 30.6 vs 43.0; and 14.3 vs 21.8). In contrast, brown foot rot was consistently most common where N was applied (mean percentages of straws affected were, respectively, 27.7 vs 10.3; 20.9 vs 1.0; and 34.3 vs 11.4). Evidence for significant effects of management regimes on eyespot was provided only by the data from Experiment 6, in which the disease was more common and severe in the early- than in the later-ploughed plots. Differences between regimes were, however, most evident in the plots given no applied N, and are, therefore, considered to be of doubtful relevance; for percentage straws with moderate or severe symptoms, the management regime × N interaction was significant at *P* = 0.02. Also in Experiment 6, brown foot rot was significantly more common in the early-ploughed and then cultivated plots than in plots testing the other management regimes (43.3 vs 16.8% straws affected in sub-plots given N) but the other two experiments provided no evidence of a similar effect.

### Experiments 8 and 9

In Experiment 8, and despite ploughing, there were, apart from the sown wheat in the set-aside/conservation covers, volunteers derived from the previous crop. They were patchy in their distribution but, in the worst-affected of the mustard-only plots, populations averaged *ca* 1 plant m^−2^. They were, therefore, sufficiently common to blur, but mostly not to obscure, the intended differences between the treatments. In Experiment 9, few naturally occurring volunteers were seen. In both experiments, most of the mustard was killed during the winter by frost but green cover was maintained by arable weeds.

Bioassays of soil sampled at the start of the treatment (i.e. set-aside) years, in late August/early September, suggested that there was less take-all inoculum in the soil going into Experiment 8 than into Experiment 9 (2.0 and 4.4% roots infected, respectively). In contrast, incidence of take-all on volunteers (sampled from plots sown at 50 plants m^−2^ and above) was greater in Experiment 8 than in Experiment 9 (respectively, 32.5 and 10.1% plants affected in spring; 46.4 and 8.9% plants affected in summer). There was no evidence that incidence or mean severity of take-all on the volunteers was affected by increasing population density from 50 to 400 plants m^−2^ but results of bioassays, in both spring and summer, indicated that amounts of take-all inoculum in the soil increased as population density increased. Accordingly, in analyses of these and other data, the variation attributed to alterations in population density was partitioned to determine the significance of the linear responses (on the square root of population density). Amounts of take-all inoculum measured using soil samples taken just before ‘later’ ploughing and of take-all disease in the following, first, test crops of winter wheat were also affected by time of ploughing. There was, however, no evidence of consistent interactions between population density and time of ploughing so only the main effects are shown in Tables 10 and 11.

**Table 10:** Take-all infectivity, measured in a bioassay, of soil sampled in summer from set-aside/conservation covers testing different populations of wheat that were ploughed at different times, and amounts of take-all and grain yield measured in the following, first, test crops of winter wheat (Experiment 8)

	Infectivity of Soil (% roots)^a^	Take-All in Spring		Take-All in Summer		Grain Yield (t ha^−1^)
		Total (% plants)	No. Roots (plant^−1^)	Total (% plants)	Take-All Index (0–100)	
Wheat plants m^−2^						
0	−2.71 (0.0)	−2.11 (1.0)	0.01	−2.63 (0.0)	0.0	9.44
4	−1.68 (2.8)	−2.03 (1.2)	0.01	−2.10 (1.0)	1.4	10.05
9	−1.68 (2.8)	−1.80 (2.2)	0.07	−1.71 (2.7)	2.4	10.02
50	−1.70 (2.7)	−1.34 (5.9)	0.11	−0.90 (13.6)	7.1	9.70
200	−0.80 (16.4)	−0.77 (17.1)	0.31	−0.64 (21.1)	11.2	9.74
400	0.00 (49.5)	−0.19 (40.3)	0.64	+0.08 (53.4)	25.7	9.50
SED (57 d.f.)	0.378	0.210	0.091	0.238	3.05	0.482
Plough date						
Early	−2.14 (0.9)	−1.55 (3.8)	0.10	−1.57 (3.7)	5.6	9.68
Later	−0.72 (18.7)	−1.20 (7.9)	0.28	−1.07 (10.1)	10.3	9.81
SED (57 d.f.)	0.218	0.121	0.053	0.137	1.76	0.277

a Data expressed as percentages were transformed before analysis and are shown as logit means with, in parentheses, the corresponding percentage values obtained by back transformation.

**Table 11:** Take-all infectivity, measured in a bioassay, of soil sampled in summer from set-aside/conservation covers testing different populations of wheat that were ploughed at different times, and amounts of take-all and grain yield measured in the following, first, test crops of winter wheat (Experiment 9)

	Infectivity of Soil (% roots)^a^	Take-All in Spring		Take-All in Summer		Grain Yield (t ha^−1^)
		Total (% plants)	No. Roots (plant^−1^)	Total (% plants)	Take-All Index (0–100)	
Wheat plants m^−2^						
0	−3.00 (0.0)	−2.27 (0.6)	0.00	−1.81 (2.1)	2.5	6.95
4	−2.65 (0.0)	−1.74 (2.5)	0.09	−1.46 (4.7)	3.7	7.37
9	−2.77 (0.0)	−1.70 (2.7)	0.07	−0.66 (20.6)	15.1	7.08
50	−2.58 (0.1)	−1.43 (4.9)	0.18	−0.36 (32.4)	22.4	6.76
200	−2.10 (1.0)	−0.59 (23.2)	0.49	+0.67 (78.9)	56.5	4.74
400	−1.34 (5.9)	−0.15 (41.9)	0.79	+0.77 (81.8)	64.1	4.87
SED (57 d.f.)	0.265	0.239	0.148	0.297	5.21	0.621
Plough date						
Early	−2.65 (0.0)	−1.42 (5.1)	0.23	−0.75 (17.9)	22.4	6.84
Later	−2.16 (0.8)	−1.21 (7.7)	0.32	−0.20 (39.5)	32.3	5.75
SED (57 d.f.)	0.153	0.138	0.086	0.171	3.01	0.358

a Data expressed as percentages were transformed before analysis and are shown as logit means with, in parentheses, the corresponding percentage values obtained by back transformation.

In both Experiment 8 and Experiment 9, there were consistent, and very significant (*P* < 0.001) positive linear effects of increasing volunteer population density on amounts of take-all inoculum in the soil into which the first test crops were sown and on the consequent incidence and severity of take-all disease (Tables 10 and 11). Test crops in Experiment 9 were evidently sown into soil that contained less inoculum than were those in Experiment 8 (reflecting differences in amounts of take-all on the volunteers) but incidence and severity of take-all measured in the following spring were similar in the two experiments. In Experiment 8, symptoms of take-all in summer were mostly slight, even in the worst-affected plots. In contrast, the disease became much more severe in Experiment 9 with moderately severe symptoms in the worst-affected plots, suggesting that conditions were more favourable for the development of take-all in 1997 than in 1996. There was consistently, and usually significantly, less take-all inoculum and disease in the early- than in the later-ploughed plots. There was no evidence that yields of grain in Experiment 8, which averaged 9.74 t ha^−1^, were affected by either population density or ploughing date. In Experiment 9, mean yield of grain was only 6.29 t ha^−1^, reflecting the greater severity of take-all than in Experiment 8, and there was a very significant (*P* < 0.001)negative linear effect of population density. Early-ploughed plots in Experiment 9 yielded significantly more grain than the later-ploughed (+1.09 t ha^−1^). Wheat that followed mustard ploughed early yielded 3.36 t ha^−1^ more than wheat that followed the largest population of volunteers ploughed late (7.87 vs 4.51 t ha^−1^). Effects on grain yield in Experiment 9 were, at least in part, explained by significant effects of both population density and ploughing date on thousand-grain weights (not shown).

Incidence and severity of take-all were greater in the second test crops than in the first but in Experiment 8 the disease only became moderately severe. In that experiment, there were again consistent and very significant (*P* < 0.001) positive linear effects of increasing volunteer population density on the disease in both spring and summer (Table 12). In Experiment 9 (Table 13), there was strong evidence of linear effects in spring (*P* = 0.003 and 0.004, respectively, for mean percentages of plants affected and mean numbers of affected roots per plant) but by summer, when the disease had become severe, the linear effects were significant at only *P* = 0.04–0.05. Early-ploughed plots in Experiment 8 had less take-all than the later-ploughed but only in spring were the differences significant; take-all was unaffected by ploughing date in Experiment 9. Despite the clear effects of ‘volunteer’ population densities on take-all in Experiment 8, there was no evidence of a consequent effect on grain yield. Grain yields in Experiment 9 averaged only 3.91 t ha^−1^, reflecting the severity of take-all, but there was, nevertheless, as in the first wheat, a very significant (*P* = 0.002) negative linear effect of volunteer population density.

**Table 12:** Take-all and grain yield measured in second test crops of winter wheat that followed set-aside/conservation covers that tested different populations of wheat and were ploughed at different times (Experiment 8)

	Take-All in Spring		Take-All in Summer		Grain Yield (t ha^−1^)
	Total (% plants)^a^	No. Roots (plant^−1^)	Total (% plants)	Take-All Index (0–100)	
Wheat plants m^−2^					
0	−1.75 (2.4)	0.04	−0.99 (11.7)	7.1	7.22
4	−1.23 (7.4)	0.23	−1.21 (7.7)	5.4	7.48
9	−0.72 (18.7)	0.33	−0.66 (20.6)	11.4	6.89
50	−0.31 (34.4)	0.60	−0.28 (35.7)	21.4	6.47
200	−0.18 (40.6)	0.63	−0.04 (47.5)	25.7	6.31
400	+0.14 (56.5)	0.90	+0.22 (60.3)	32.8	6.78
SED (57 d.f.)	0.207	0.134	0.258	4.53	0.766
Plough date					
Early	−0.87 (14.5)	0.34	−0.60 (22.8)	16.1	6.77
Later	−0.48 (27.1)	0.57	−0.39 (30.8)	18.5	6.95
SED (57 d.f.)	0.120	0.077	0.149	2.62	0.442

a Data expressed as percentages were transformed before analysis and are shown as logit means with, in parentheses, the corresponding percentage values obtained by back transformation.

**Table 13:** Take-all and grain yield measured in second test crops of winter wheat that followed set-aside/conservation covers that tested different populations of wheat and were ploughed at different times (Experiment 9)

	Take-All in Spring		Take-All in Summer		Grain Yield (t ha^−1^)
	Total (% plants)^a^	No. roots (plant^−1^)	Total (% plants)	Take-All Index (0–100)	
Wheat plants m^−2^					
0	+0.83 (83.6)	3.2	+1.10 (89.5)	70.3	4.68
4	+1.04 (88.4)	3.8	+1.03 (88.2)	67.6	4.78
9	+1.42 (94.0)	4.6	+1.24 (91.7)	72.2	4.12
50	+1.46 (94.4)	5.0	+1.19 (91.0)	73.6	3.77
200	+1.70 (96.3)	4.6	+1.49 (94.7)	78.5	3.03
400	+1.59 (95.5)	5.7	+1.57 (95.4)	80.3	3.07
SED (57 d.f.)	0.272	0.75	0.306	7.11	0.690
Plough date					
Early	+1.22 (91.5)	3.8	+1.22 (91.5)	74.8	4.18
Later	+1.46 (94.4)	5.1	+1.32 (92.8)	72.8	3.63
SED (57 d.f.)	0.157	0.44	0.177	4.10	0.398

a Data expressed as percentages were transformed before analysis and are shown as logit means with, in parentheses, the corresponding percentage values obtained by back transformation.

Third test crops in Experiment 8 (Table 14) had more take-all than did the second test crops but the disease was still not as severe as it had been in the second test crops in Experiment 9. In the latter experiment, the third test crops had less take-all than did the second, presumably as a consequence of TAD. There was little evidence, in either experiment, that take-all in the third wheats was affected by volunteer populations but in Experiment 9, incidence and severity of the disease were significantly greater in the later-ploughed than in the early-ploughed plots.

**Table 14:** Take-all and grain yield measured in third test crops of winter wheat that followed set-aside/conservation covers that tested different populations of wheat and were ploughed at different times (Experiments 8 and 9)

	Experiment 8		Grain Yield (t ha^−1^)	Experiment 9		Grain Yield (t ha^−1^)
	Take-All in Summer			Take-All in Summer		
	Total (% plants)^a^	Take-All Index (0–100)		Total (% plants)	Take-All Index (0–100)	
Wheat plants m^−2^						
0	+0.40 (68.4)	36.6	7.07	−0.35 (32.7)	19.6	6.62
4	+0.77 (81.9)	45.6	7.31	−0.21 (39.3)	22.2	7.27
9	+1.04 (88.3)	44.3	7.15	+0.02 (50.5)	29.4	7.03
50	+0.82 (83.2)	40.3	6.98	−0.25 (37.4)	21.7	7.02
200	+0.85 (84.0)	42.9	7.19	+0.04 (51.5)	31.8	5.99
400	+0.98 (87.1)	46.3	7.49	−0.09 (45.1)	26.6	6.57
SED (57 d.f.)	0.351	8.14	0.346	0.185	6.13	0.392
Plough date						
Early	+0.94 (86.2)	46.3	7.06	−0.26 (36.9)	22.3	6.77
Later	+0.68 (79.0)	39.1	7.34	−0.02 (48.5)	28.1	6.73
SED (57 d.f.)	0.203	4.70	0.200	0.107	3.54	0.226

a Data expressed as percentages were transformed before analysis and are shown as logit means with, in parentheses, the corresponding percentage values obtained by back transformation.

There was evidence in both experiments of linear effects of volunteer population density on the incidence of eyespot in the first test crops but while the relationship was positive in Experiment 8 (*P* = 0.005) it was negative in Experiment 9 (*P* = 0.002). There was evidence of similar, negative, relationships in the eyespot data from the second and third test crops in Experiment 9 but not in Experiment 8.

### Experiments 10 and 11

The fourth consecutive crop of wheat that preceded Experiment 10 had severe take-all, with a TAI, in summer, of about 90. On average, 91.8% of wheat plants sampled from the wheat plots or from the set-aside/conservation covers in the following spring (before the latter had been topped or sprayed with glyphosate) were affected by the disease, with, on average, 3.9 infected roots plant^−1^. In summer, the continuous wheat had a TAI of 28.3 suggesting that TAD had, as expected, established. There was a tendency for there to be more take-all on plants sampled from the set-aside/conservation covers (Table 15) but as the differences were not significant, and the plants were sampled on 19 June, only 8 days after glyphosate sprays were applied to the set-aside/conservation covers and two days before they were topped (in different treatments), it is reasonable to suspect that they were spurious. It is, however, possible that they were a consequence of sowing wheat at a smaller rate in the set-aside/conservation covers than in the wheat plots. Whether these differences were or were not entirely spurious, they contrast very markedly with the results of bioassays of soils sampled about 2 months later, which indicated that there was significantly more inoculum of the take-all fungus in the plots testing wheat than in those testing set-aside/conservation covers that had been topped or, especially, sprayed with glyphosate (67.7, 54.0 and 20.3% roots infected, respectively; *P* = 0.003). The average yield of grain from wheat plots in the first year was 5.49 t ha^−1^. In the second year of the experiment, all plots were cropped with winter wheat. Take-all in the continuous wheat was more severe than it had been in the previous year, suggesting that TAD was not yet fully effective. There were differences between the treatments in amounts of take-all, in both spring (not shown) and in summer (Table 15), that reflected the evident differences in the amounts of inoculum to which they were exposed. Differences between the wheat after wheat and the wheat after set-aside/conservation covers that had been topped were, however, relatively small, and mean grain yield from the latter was only 0.37 t ha^−1^ more than that from the wheat after wheat (Table 15). In contrast, wheat after set-aside/conservation covers that had been sprayed with glyphosate gave a mean yield that was 2.28 t ha^−1^ greater than that of wheat after wheat. In the third year of the experiment, amounts of take-all in plots after wheat and in plots after set-aside/conservation covers that had been topped differed relatively little but there was significantly more, especially in summer, in plots after set-aside/conservation covers that had been sprayed with glyphosate (Table 15). Correspondingly, grain yields from the latter were, on average, 0.84 t ha^−1^ smaller than those from the continuous wheat.

**Table 15:** Amounts of take-all on wheat plants sampled in summer from crops ofwinterwheat or from set-aside/conservation covers in three consecutive years, and yields of grain obtained from the wheat crops (Experiment 10)

Sequence^a^	Year 1		Year 2			Year 3		
	Take-All		Take-All		Grain Yield(t ha ^−1^)	Take-All		Grain Yield(t ha^−1^)
	Total (% plants)^b^	Take-All Index (0–100)	Total (% plants)	Take-All Index (0–100)		Total (% plants)	Take-All Index (0–100)	
WW/WW/WW	+0.67 (78.7)	28.3	+1.56 (95.3)	78.2	5.32	−0.09 (44.9)	23.2	7.96
ST/WW/WW	+1.25 (91.9)	37.6	+1.15 (90.4)	58.3	5.69	−0.01 (49.1)	25.7	7.48
SG/WW/WW	+1.35 (93.2)	39.6	+0.42 (69.1)	34.4	7.60	+0.53 (73.8)	43.2	7.12
SED (6 d.f.)	0.358	5.21	0.452	12.07	0.516	0.139	5.83	0.152

a WW>winter wheat; ST>set-aside/conservation covers, topped as necessary; SG>set-aside/conservation covers, sprayed with glyphosate. See text for further details.

b Data expressed as percentages were transformed before analysis and are shown as logit means with, in parentheses, the corresponding percentage values obtained by back transformation.

The winter wheat that preceded Experiment 11, which was the second of two consecutive crops, had only slight take-all, with a TAI of about 14. In the following spring, 44.1% of wheat plants sampled from the wheat plots or from the set-aside/conservation covers were affected by the disease, with, on average, 1.8 infected roots plant^−1^. In summer, take-all was more severe than it had been in the preceding year but there were only small, and not significant, differences between plants sampled from plots testing wheat and from those testing set-aside/conservation covers (Table 16). Plots of wheat gave a mean grain yield of only 3.59 t ha^−1^. In bioassays of soil samples collected after harvest, the mean percentage of roots affected by take-all was 70.1 and there were, again, no significant differences between the treatments. The same was true of plants sampled in the spring of the following (i.e. second) year but in summer, when take-all was severe, symptoms were significantly less severe on plants sampled from plots testing set-aside/conservation covers than on those from plots testing wheat (after wheat or after set-aside/conservation covers in the first year only). Differences between the treatments in the percentages of plants affected by take-all were relatively small (albeit very significant; *P* < 0.001), and the proportionately bigger differences between TAIs were mostly attributable to differences in percentages of plants with severe symptoms (71.4, 88.0 and 93.4%, respectively, in samples from set-aside/conservation covers, and from wheat after wheat and from wheat after one year set-aside/conservation covers; *P* = 0.006). Grain yields from wheat with such severe take-all were, predictably, small (Table 16). In the third year, percentages of plants affected by take-all in spring were smaller in plots of continuous wheat than in those testing first or second wheats after set-aside/conservation covers (17.8, 39.0 and 43.0, respectively) but the differences were not significant. In summer, symptoms of take-all were consistently less severe than they had been in the previous year, almost certainly as a consequence of TAD. There was, however, significantly more take-all in first wheats than in second wheats after set-aside/conservation covers or in continuous wheats; differences between the latter two treatments were small and not significant (Table 16). The much larger mean TAI for the first than for the second wheats after set-aside/conservation covers or for the continuous wheats was partly a consequence of a larger mean percentage of plants affected by the disease (Table 16) but could mostly be attributed to very large, and significant, differences in percentages of plants with severe symptoms (68.6, 28.2 and 34.5%, respectively; *P* = 0.006). The greater severity of take-all in the first wheats was reflected in their grain yields, which were, on average, about 1.4 t ha^−1^ smaller than the mean for the other two treatments.

**Table 16:** Amounts of take-all on wheat plants sampled in summer from crops of winter wheat or from set-aside/conservation covers in three consecutive years, and yields of grain obtained from the wheat crops (Experiment 11)

Sequence^a^	Year 1^b^		Year 2		Grain Yield (t ha^−1^)	Year 3		Grain Yield (t ha^−1^)
	Take-All		Take-All			Take-All		
	Total (% plants)^c^	Take-All Index (0–100)	Total (% plants)	Take-All Index (0–100)		Total (% plants)	Take-All Index (0–100)	
WW/WW/WW	+0.44 (70.2)	37.9	+2.10 (98.0)	95.9	0.98	+0.67 (78.6)	53.1	7.10
ST/WW/WW	+0.23 (60.9)	34.5	+2.08 (98.0)	97.7	1.53	+0.95 (86.5)	53.8	6.63
ST/ST/WW	—	—	+1.70 (96.3)	90.3	—	+1.78 (96.7)	82.6	5.50
SED (6 d.f.)^d^	0.366	11.74	0.053	1.36	0.199	0.251	7.02	0.474

a WW>winter wheat; ST>set-aside/conservation covers, topped as necessary. See text for further details.

b The two sequences testing set-aside/conservation covers (the second and third in the list) were identically treated in Year 1, so the figures presented are means for the two treatments.

c Data expressed as percentages were transformed before analysis and are shown as logit means with, in parentheses, the corresponding percentage values obtained by back transformation.

d Except 3 d.f. for grain yield in Year 2.

## Discussion

The work described in this article was designed mainly to investigate further the effects, on take-all inoculum and disease, of wheat volunteers growing in break crops or set-aside/conservation covers that interrupted sequences of winter wheat, and of controlling them at different times during the growing season. Two of the experiments were, though, designed to study the development of take-all in sequences of winter wheat grown after different break crops in which volunteers were, as far as possible, controlled. Despite the relatively narrow focus of these experiments, they illustrate well the unpredictable nature of take-all epidemics.

We obtained no convincing evidence (from Experiments 1 and 2) that amounts of take-all in sequences of winter wheat are influenced to any significant extent by growing different break crops, which confirms earlier results reported by Prew & Dyke (1979). This is despite evidence that OSR, specifically, can be infected to a limited extent by the take-all fungus (Kollmorgen *et al*., 1983). Significantly greater yields in Experiment 1 from first wheat after oats than from first wheats after other break crops were not associated with differences in take-all, and were attributed to differences in lodging. In Experiment 2, second wheats after oats had, on average, less take-all and gave larger yields, than second wheats after non-graminaceous break crops but this result is considered to be spurious because it could be attributed to data from just one of the four replicate plots.

These results do not, however, preclude the possibility of indirect effects on take-all of growing different break crops, as a consequence of, for example, differences in the opportunities that they provide for the early sowing of following crops of wheat, and the risks that that implies. Severity of take-all in first wheats might also be affected by differences in the amounts of residual nitrogen left in the soil after growing different break crops. The direct effects of applied N on the severity of take-all are illustrated by the results from our Experiments 5, 6 and 7, in which take-all in the first wheats was consistently less severe where N had been applied than where it had not. The differences between the two N rates tested (none vs a standard rate) were deliberately extreme (for reasons explained above) but the results are consistent with those from previous experiments that tested more, including more agronomically relevant, rates (Widdowson *et al*., 1985). More relevant to this discussion, however, are the results obtained from second wheats in Experiment 5, which had less take-all where N had been applied to first wheats than where it had not. Second wheats in Experiments 6 and 7 did not, though, show similar effects of N applied to the previous crop. The two experiments that tested the effects on take-all of volunteers growing in OSR (Experiments 3 and 4) also included a test of nitrogen. There was evidence from one of them (Experiment 4) that amounts of inoculum in the soil after the rape had been harvested was greater where the smaller of two rates of nitrogen had been applied to the crop than where the larger amount had been applied. Although this was not reflected in the amounts of take-all seen when the first wheats were sampled in spring, take-all in summer was much more prevalent in plots that followed OSR that contained volunteers that were not destroyed and were given the smaller of the two nitrogen rates than in all other treatment combinations.

Different break crops may also harbour different populations of take-all-susceptible cereal volunteers, either because of differences in the ease with which they can be controlled or in the perceived need to do so. These undoubtedly have the potential to maintain the take-all fungus during the cultivation of non-susceptible break crops but whether they pose a significant and consistent risk to following susceptible crop(s) is uncertain. It can, however, be expected that 1-year set-aside/conservation covers that interrupt sequences of take-all susceptible cereals will often pose a bigger threat to following susceptible cereals than do 1-year break crops. There are three main reasons for this. First, populations of cereal volunteers are typically very much greater in the former, and especially if green cover is provided by natural regeneration; second, there are likely to be restrictions on how, and how early, covers can be destroyed; and third, prohibited application of N may, as discussed above, also increase the risk.

The potential for volunteers to influence amounts of take-all in following, first, wheat crops is well demonstrated by results from four of the experiments that we describe. In Experiments 3 and 4, wheat (to simulate volunteers) was sown with an OSR break crop that was conventionally managed apart from the herbicide treatments that the experiments were designed to test. In both experiments, soil sampled after the rape had been harvested contained most inoculum, and following crops of winter wheat had more take-all, where volunteers in the rape had not been destroyed than where they were not present. Although differences between individual treatments were not always significant, amounts of inoculum after the rape and of take-all in the following wheat tended to be decreased by destroying the volunteers (compared to volunteers not destroyed), and early destruction tended to be more effective than later destruction. Very similar results were obtained from Experiments 8 and 9, which tested effects of wheat (to simulate volunteers) that was sown with mustard, and was managed in ways that were appropriate for set-aside/conservation covers. In these, amounts of inoculum to which the first test crops of wheat were exposed, and amounts of take-all that developed on the roots of the wheat, were consistently, and usually significantly, less where the covers were ploughed early than where they were ploughed late. These experiments also demonstrated convincingly that there was a very close positive relationship between numbers of volunteers in the covers and the consequent amounts of take-all inoculum to which the following crops of wheat were exposed, and the incidence and severity of the disease that subsequently developed on their roots. Using the square roots of numbers of volunteers per m^2^ in these analyses makes good biological sense because they correspond to differences in the linear distances between them. These data show that, especially within experiments, and as previously reported by Gutteridge & Hornby (2003), differences in the relative amounts of inoculum in the soil, estimated using bioassays, can be good predictors of differences in the likely incidence and severity of take-all in the following crop. Differences between sites and seasons in the relative amounts of inoculum detected do not, however, provide a reliable guide to the relative amounts of take-all that can be expected to develop on the roots of the following crops. This is well illustrated by the results of our Experiments 8 and 9, which were in different fields and were started in successive years. More inoculum was detected after set-aside/conservation covers in Experiment 8 than in Experiment 9 but take-all in the following crops of winter wheat, measured in summer, was more severe in Experiment 9 than in Experiment 8.

It is, thus, clear from these results that amounts of take-all in first wheats are likely to increase with increasing numbers of volunteers in the preceding break crop or cover and also with increasing delays in their destruction. Despite this, symptoms of take-all in our experiments, assessed in summer, were mostly slight. The principal exception was Experiment 9 but even here it was only in plots testing covers with the two largest populations of wheat (200 or 400 plants m^−2^) that symptoms became moderately severe, and especially in those that were later-ploughed, which evidently agrees with the effects of natural regeneration set-aside reported by Yarham & Gladders (1993). Not surprisingly, perhaps, it was also Experiment 9 that provided the best evidence for associated effects on grain yield. There was also, though, in Experiment 3, and despite slight take-all, a slighter larger yield of grain from wheat after rape that contained no volunteers than after rape that contained volunteers that were not destroyed or were destroyed late.

In Experiments 5, 6 and 7, where covers were established by natural regeneration, effects of destroying them at different times (using cultivations and/or herbicides) were much less evident. In Experiment 5, there was, however, much more take-all in the blocks with much vegetation than in those with little vegetation, which is consistent with the effects of different numbers of volunteers seen in Experiments 8 and 9. Importantly, though, there was nothing in the results of Experiments 5, 6 and 7 that seriously contradicted the results of the other experiments. We attribute our failure to detect significant effects of the treatments tested in these experiments mostly to the variability in the extent and composition of the vegetation that grew in the covers, which emphasises the benefits of imposing much greater control over the treatments tested in Experiments 8 and 9, despite requiring much greater effort. Differences between treatments, and between plots, in mean numbers of volunteers, and also patchiness in their distribution, are likely to have been particularly important. It is possible, however, that the grasses that grew in these experiments (that were also variable within and between experiments) might have influenced the development of the take-all (Gutteridge *et al*., 2006), and might also, therefore, have contributed to variability in its incidence and severity.

While the risk of damaging take-all in crops of susceptible cereals grown immediately after non-graminaceous break crops (and often, as we have shown, after set-aside/conservation covers) is very small, the risk does become very much greater if cereals are grown consecutively. In winter wheat grown in the UK, severe take-all is now often seen in second crops (and has been since the 1980s) whereas in earlier years second wheats were less often severely infected (Hornby *et al*., 1998); formerly it was third or fourth wheats that were usually considered to be most at risk. Changes in the management of the second wheats themselves have probably contributed to this, especially early sowing, which is one of the biggest risk factors. This is mainly because the infectivity of colonised crop debris decreases as it decomposes, and because infection is favoured by warm soils (Hornby, 1975). Another potentially important determinant of disease severity at harvest is the amount of initial inoculum of the take-all fungus left in the soil by one crop to which the following crop may be exposed (Hornby *et al*., 1998). Consequently, the increased frequency of severe take-all in second wheats might also be explained, in part, by their exposure to greater concentrations of inoculum generated during the cultivation of the preceding first wheats. Changes in the management of the first wheats themselves are likely to have had the largest effects and, therefore, be the most important. The direct effects on take-all of different sowing dates, for example, are well documented but there is also good evidence for both wheat (Gutteridge & Hornby, 2003) and barley (Jenkyn *et al*., 1992) that they can have continuing, albeit smaller, residual effects on the severity of take-all in the following crops. Small differences in amounts of non-damaging take-all in first wheats, perhaps resulting from variations in the management of preceding breaks or covers, might also, therefore, be important if they were to generate corresponding differences in the more severely affected crops that follow them. Despite the assumed importance of initial inoculum concentrations, it was, however, acknowledged by Hornby *et al*. (1998) that they are not always correlated with the disease at harvest, and also that small amounts of infection in 1 year can give rise to severe disease in the following year. In experiments reported by Jenkyn *et al*. (1998), for example, there was, as expected, more, albeit slight, take-all in winter wheat where wheat volunteers had been allowed to develop in preceding set-aside covers than where they had been controlled by ploughing (before sowing cover crops) but these differences were not reflected in the much more severe take-all that ultimately developed in the following (second) wheats. In all of the experiments reported here we, therefore, grew at least two test crops so that we could investigate further these relationships between take-all in consecutive (especially first and second) crops of wheat.

With the exception of Experiments 5, 6 and 8, to which we return later, take-all was very much more severe in second wheats after break crops or set-aside/conservation covers (with mean take-all indices (TAIs) ranging from 72.3 to 91.7) than it had been in the first wheats (with TAIs ranging from 0.9 to 29.9 but in different experiments), with correspondingly small, and commercially unacceptable, grain yields. Differences in the severities of take-all in the second wheats in these experiments were not related to their sowing dates, which were all between 23 and 25 September. Neither was there any clear correlation between the severities of take-all in the two successive crops. Indeed, take-all in second wheats was most severe (i.e. TAIs of 90 or more) where take-all in first wheats was either least severe (Experiments 2 and 4; TAI = 0.9 and 1.7, respectively) or where it was most severe (Experiment 7; TAI = 29.9; data from plots to which N had been applied when growing first wheats). Similarly, amounts of take-all inoculum in the soil after first wheats had been harvested (not measured in all experiments) were evidently not a reliable guide to the relative risk of severe take-all in the following crop. These experiments also provided little evidence that differences in the severities of take-all in the second wheats were directly related to differences in the severities of take-all seen in first wheats, as a consequence of the treatments imposed on the break crops or set-aside/conservation covers that preceded them. Indeed, in Experiment 3, take-all was less severe where volunteers in the rape were not destroyed or were destroyed late than where they were absent or destroyed early, which was the opposite effect to that seen in the first wheats. Similar negative correlations between the severities of take-all in consecutive crops have been reported previously in, for example, experiments testing fungicides, and have been tentatively attributed to differences in the potency of TAD in the second of two crops as a consequence of differences in disease in the first (Bateman *et al*., 2004). The different management regimes tested in Experiment 7 had no clear effects on take-all in the first wheats so it is not surprising that none were seen in the data from the second wheats. In contrast, the treatments tested in Experiment 9 (different populations of volunteers in, and different times of ploughing of, set-aside/conservation covers) had clear and very significant effects on the severity of take-all in the following wheat. Despite this, severity of take-all in the second wheat was relatively little affected by the treatments, especially in summer; date of ploughing had no effect and the small effect of different populations of volunteers could mostly be attributed to slightly more severe take-all following covers with the two largest, and agronomically atypical, populations. The significant negative effects of the different populations of volunteers on the yields of grain obtained from the second wheats can, perhaps, be attributed to the somewhat clearer effects of the treatments earlier in the season.

Second wheats in Experiments 5, 6 and 8 had much less severe take-all than second wheats in the experiments discussed above, with mean TAIs of 26.7, 5.3 and 17.3, respectively (data for Experiments 5 and 6 are from plots to which N had been applied when growing first wheats). This can almost certainly be attributed, in large part, to the fact that they were later sown (on 30 September, and 16 and 9 October, respectively); none of these dates are, however, considered to be unusually late. Coincidentally they also followed first wheats that were later sown than first wheats in most of the other experiments (on 28 September, 26 September and 10 October, respectively). First wheats in the other experiments were sown between 17 and 25 September except in Experiment 7 (3 October). These three experiments, and especially Experiment 8, also provided somewhat stronger evidence for positive associations between amounts of take-all in first and second wheats (negative correlations can also occur, as a consequence of TAD). Thus, in second wheats in Experiment 5 there was, as in the first wheats, more take-all in the blocks that had most green cover in the set-aside year than in those that had little cover. In this experiment, also, there was less take-all in the second wheats where N had been applied to the first wheats than where it had not, which reflected the effect of N in the first wheats. It was, however, Experiment 8 that provided the clearest evidence that management of set-aside/conservation covers can, in some circumstances, have very significant effects on take-all in the second of two following crops of wheat. Thus, amounts of take-all in the second wheats were positively, and very significantly, related to numbers of volunteers in the set-aside/conservation covers 2 years before. There was also less take-all in the second wheats where the set-aside/conservation covers had been ploughed early than where they had been ploughed later but the effect was significant only in spring. It should be noted that although there was more take-all on the roots of first wheats in Experiment 9 than on those in Experiment 8, this does not provide an adequate explanation for the different effects of treatments on second wheats in the two experiments because there was considerable overlap between them in the disease data obtained from first wheats. Apart from Experiments 10 and 11, third wheats were tested only in Experiments 8 and 9, which provided no evidence for effects on take-all of numbers of volunteers in the set-aside/conservation covers grown 3 years before. The apparent residual effect of ploughing date on severity of take-all on the roots of third wheats in Experiment 9 is probably spurious because it evidently had no effect on the severity of take-all in the preceding crop.

Our results confirm, therefore, that early-sown second wheats can be expected to suffer, almost invariably, from very severe take-all. They also suggest that differences in the amounts of inoculum to which such second wheats are exposed as a result of differences in the management of preceding crops and covers are, consequently, unimportant. In contrast, second wheats in experiments that were (not deliberately) sown 1–3 weeks later had, on average, much less take-all, and its severity evidently did reflect differences in the management of the preceding crops and covers. To confirm this would require designed experiments in which second wheats, sown on a wider range of dates than in the experiments reported here, followed first wheats that left different amounts of inoculum in the soil, either as a consequence of varying the management of a preceding crop or cover or as a result of exposing the first wheats to different amounts of artificial inoculum of the take-all fungus.

As noted above, second wheats in Experiments 1–9 that were sown between 23 and 25 September all had severe take-all, and the relatively small differences between them were unrelated to the severity of the disease in the preceding crop. This is consistent with results from other experiments at Rothamsted, and with the assertion by Hornby *et al*. (1998) that assessment of take-all in a crop does not reliably indicate the risk to a following crop, but it is something that is often overlooked. There is, however, an important exception. When severe take-all is seen in a crop, it can almost be guaranteed that symptoms in the following crop will be much less severe, as a consequence of TAD. In our experiments, a clear example of this phenomenon was in Experiment 1 where second test crops of wheat grown after wheat in the treatment (i.e. break crop) year, which were the fifth consecutive crops of wheat, had much less severe take-all, and gave much larger yields of grain, than second wheats after break crops *per se*. Grain yields from crops of wheat grown in fields exhibiting effective TAD will never match those from first wheats grown after a break but there are circumstances where growing long runs of wheat (to exploit TAD) makes good commercial sense (e.g. in fields with difficult access or with difficult soils that limit alternative cropping options). In order to generate effective TAD it is not, however, sufficient just to grow a long sequence of susceptible crops; it is necessary for one or more of those crops to have had severe take-all (Hornby & Gutteridge, 1995). Furthermore, TAD generated under very long sequences of spring barley, in which take-all is typically less severe than in comparable crops of wheat, is evidently not sufficiently potent to protect subsequent crops of winter wheat (Gutteridge *et al*., 1996). Against this background, it is not surprising that some growers have asked whether effective TAD can be generated if set-aside/conservation covers (containing large populations of wheat) are grown during the year(s) when the risk of severe take-all is greatest, and, just as importantly, if effective TAD will be maintained if long sequences of wheat crops are interrupted by one or more years of set-aside. It is these questions that Experiments 10 and 11 were designed to address (although the number of treatments that we could test, and the duration of the experiments, were limited by the available resources). The answers to the questions are not self-evident. For example, growing wheat in combination with other species (as in set-aside conservation covers) may affect the development of take-all, and TAD (e.g. Gutteridge *et al*., 2006). Also, while roots of wheat in set-aside/conservation covers to which N has not been applied might be expected to have more severe take-all than roots of a wheat crop to which N has been applied, amounts of biomass in the former, both above and below ground, can, as a result of insufficient N, be expected to be much smaller as well so it is difficult to predict the likely effects on total amounts of diseased tissue, and on amounts of inoculum to which the following crop is exposed.

In Experiment 10, the effects of set-aside/conservation covers were tested on a site where severe take-all had already developed, and where it was assumed that TAD had established. Data obtained in the first (treatment) year from plots testing continuous wheat suggested that that was the case but in the following year there was resurgence in take-all suggesting that fully effective TAD had not yet been established. Where, in the first year of the experiment, covers were destroyed relatively early, using sprays of glyphosate, much less inoculum was generated than where covers were not destroyed until the plots were ploughed in readiness for sowing the following test crops of wheat. That wheat consequently had less take-all, and gave significantly larger yields, than the continuous wheat, which is consistent with the results of other experiments that we describe. This decrease in severity of take-all evidently, however, disrupted TAD because in the next year the second wheat after the glyphosate-treated covers had significantly more take-all, and gave significantly smaller yields, than the continuous wheat. First and second wheats after covers that were not destroyed similarly had less and more take-all, respectively, than the continuous wheat, and gave larger and smaller yields. The differences were, however, relatively small, and total grain yields over the 2 years differed little, being 13.17 and 13.28 t ha^−1^, respectively, for the wheat after covers and the continuous wheat.

Experiment 11, in contrast to Experiment 10, was on a site where take-all had not become severe, and where effective TAD was not expected to have established. In the third year of the experiment, wheat that followed 2 years in set-aside/conservation covers had more take-all, and gave smaller yields, than the continuous wheat, suggesting that there were unacceptably large effects of the covers on the disease, and on the development of TAD. There was, however, as in Experiment 10, relatively little difference between wheat that was grown without interruption and that which was interrupted with only a 1-year set-aside/conservation cover. The intention in this experiment was to substitute set-aside/conservation covers for wheat in the year when take-all was expected to be most severe, i.e. in the first year of the experiment, when the field was in its third consecutive crop of wheat. In this we failed because it was the following, fourth, crop that had very severe take-all (and correspondingly small yields) illustrating, again, that it is not easy to predict take-all, and especially how quickly it will develop. Substituting a set-aside/conservation cover for wheat means, of course, that grain yield in that year is forfeited but that is not a serious concern if covers coincide with the stage of epidemic development when symptoms of take-all are as severe, and grain yields are as small, as they were in the second year of Experiment 11. Indeed, the rationale for interrupting, with set-aside/conservation covers, sequences of wheat that are intended to exploit TAD is that, under current arrangements, the support payments for such covers, in combination with small inputs, are likely to generate positive financial returns whereas expensive-to-grow but low-yielding crops of wheat will not. From our results, we tentatively conclude that interrupting, with set-aside/conservation covers, long sequences of winter wheat, in which effective TAD has been established may not jeopardise the maintenance of that TAD provided the covers contain adequate populations of wheat and are not destroyed prematurely. Similarly substituting covers for wheat during the build-up phase of an epidemic may be an equally viable strategy but, to gain maximum advantage, the cover needs to coincide with maximum severity of take-all, which is difficult to predict. We emphasise, however, that it would be very risky to base on-farm strategies on our results that were obtained from only two, relatively simple, experiments, carried out on a single soil type. They do, however, suggest that there is potential for exploiting set-aside/conservation covers in the ways proposed, and that further research should be considered.
